# The combination of *ACE* I/D and *ACE2* G8790A polymorphisms revels susceptibility to hypertension: A genetic association study in Brazilian patients

**DOI:** 10.1371/journal.pone.0221248

**Published:** 2019-08-20

**Authors:** Denise S. Pinheiro, Rodrigo S. Santos, Paulo C. B. Veiga Jardim, Elisangela G. Silva, Angela A. S. Reis, Gustavo R. Pedrino, Cirano J. Ulhoa

**Affiliations:** 1 Postgraduate Program in Biological Sciences, Institute of Biological Sciences, Federal University of Goiás (UFG), Goiânia, Goiás, Brazil; 2 Department of Nature Sciences (LEdoC), Special Academic Unit of Human Sciences, Federal University of Goiás (UFG), Goiás, Goiás, Brazil; 3 League of Hypertension, Faculty of Medicine, Federal University of Goiás (UFG), Goiânia, Goiás, Brazil; 4 Department of Biochemistry and Molecular Biology, Institute of Biological Sciences, Federal University of Goiás (UFG), Goiânia, Goiás, Brazil; 5 Department of Physiological Sciences, Institute of Biological Sciences, Federal University of Goiás (UFG), Goiânia, Goiás, Brazil; Unicamillus, Saint Camillus International University of Health Sciences, ITALY

## Abstract

**Background:**

Systemic arterial hypertension (SAH) is a multifactorial condition that already affects one third of the worldwide population. The identification of candidate genes for hypertension is a challenge for the next years. Nevertheless, the small contribution of each individual genetic factor to the disease brings the necessity of evaluate genes in an integrative manner and taking into consideration the physiological interaction of functions. Angiotensin I–converting enzymes, ACE and ACE2, are key regulators of blood pressure that have counterbalance roles by acting on vasoactive peptides from Renin-Angiotensin-Aldosterone System (RAAS). Insertion/deletion (I/D) polymorphism of *ACE* gene and single nucleotide polymorphism G8790A of *ACE2* gene have been associated with susceptibility to SAH, but the literature is controversial. We proposed to evaluate these two polymorphisms jointly exploring the combined effects of *ACE* and *ACE2* genotypes on SAH susceptibility, an approach that have not been done yet for *ACE* and *ACE2* polymorphisms.

**Methods and findings:**

This genetic association study included 117 hypertensive (mean age 59.7 years) patients and 123 normotensive and diabetes-free controls (mean age 57.5 years). *ACE* and *ACE2* polymorphisms were genotyped by SYBR Green real-time PCR and RFLP-PCR, respectively. Crude and adjusted odds ratio (OR) values were calculated to estimate the susceptibility to SAH development. It was obtained homogeneity regarding distribution by sex, age range, smoking, alcohol consumption and body mass index (BMI) between case and control groups. No-association was verified for each gene individually, but the combination of *ACE* and *ACE2* polymorphisms on female gender revealed a significative association for DD/G_ carriers who had a 3-fold increased risk to SAH development (p = 0.03), with a stronger susceptibility on DD/GG carriers (7-fold increased risk, p = 0.01). The D allele of *ACE* showed association with altered levels of lipid profile variables on case group (VLDL-cholesterol, p = 0.01) and DD genotype in all individuals analysis (triglycerides, p = 0.01 and VLDL-cholesterol, p = 0.01).

**Conclusion:**

These findings indicate that the combination of *ACE* and *ACE2* polymorphisms effects may play a role in SAH predisposition been the DD/G_ genotype the susceptibility profile. This result allowed us to raise the hypothesis that an increased activity of ACE (prohypertensive effects) in conjunction with reduced ACE2 activity (antihypertensive effects) could be the underlining mechanism. The association of *ACE* D allele with lipid alterations indicate that this can be a marker of poor prognostic on SAH evolution and contribute to CVD development. Although these preliminary findings must be confirmed by further researches with larger sample size, we could observe that the integrative analysis of *ACE* and *ACE2* can be an informative tool in hypertension understanding that needs to be explored in new studies.

## Introduction

Systemic arterial hypertension (SAH) is a leading cause of cardiovascular diseases (CVD) reaching the prevalence of 30% in the world population [1]. As a multifactorial condition, to tackle this disease not only the environmental factors but also the emerging weight of genetic factors need to be considered for years to come, with application on genetic risk scores development [2], once genetics can be responsible for about 40% of the interindividual variance in blood pressure levels [3]. In this sense, several research groups have dedicated to studying the genetic basis of hypertension, but it has been pointed out the small contribution of each one of the genetic factors and the interaction among them as a problem in identifying genetic markers for the disease [4].

Renin-Angiotensin-Aldosterone System (RAAS) constitute the main regulator of blood pressure and fluid-electrolyte balance. Angiotensin I-converting enzyme (ACE) and your new discovered homologue, ACE2 [5,6], are the key enzymes of this system constituting two arms that counterbalance each other in blood pressure regulation [7]. Although ACE and ACE2 present several other roles besides their functions as angitensinases [8–12], ACE main role is the conversion of angiotensin I (Ang-I) to angiotensin II (Ang-II), a potent vasoactive peptide that leads to effects mainly hypertensives, while ACE2 has predominantly opposite effects by two mechanisms: conversion of Ang-I in Angiotensin 1–9, that is converted by ACE in the vasodilator peptide Angiotensin 1–7 (Ang 1–7); and directly conversion of Ang-II in Ang 1–7 [5–7].

Polymorphisms in components of RAAS constitute the focus of the genetic association studies related to hypertension, been the insertion/deletion (I/D) polymorphism (rs4646994) of the *ACE* gene one of the most studied in this field [13,14]. *ACE* gene is located on chromossome 17q23.3 and the I/D polymorphism involves the presence/ absence of an *Alu* sequence of 287 pb in intron 16 of the gene [15]. Although there are discrepant results in the literature, findings from many studies evidence the association of D allele or DD genotype with elevated blood pressure and/or hypertension [16], as well as, CVD like stroke [17,18] and coronary heart disease [19–21], having as a probable mechanism an increased enzyme activity [15,22].

Since ACE2 enzyme was discovered [5,6], some researchers have explored the association of single nucleotide polymorphisms (SNPs) of the gene (localized on chromosome Xp22) with hypertension, with special attention to G8790A polymorphism (rs2285666) in intron 3, nevertheless this issue remains inconclusive and most of studies were conducted in China [23].

Once there are large inconsistent findings in the literature, the influence of polymorphisms in the genes *ACE* and *ACE2* on hypertension susceptibility remains a topic to be better studied and explored in different populations. Until now, most previous studies have focused on the association between ACE or ACE2 polymorphisms and hypertension individually. Here we proposed to investigate the association of polymorphisms of the two key enzymes of blood pressure regulation with SAH susceptibility exploring their interaction effects by the genotypic combination analysis. This approach has not been observed in any other study involving ACE and ACE2 polymorphism so far and we have obtained appreciable results that can help the discussion about the role of the collaborative function of those enzymes in disease pathogenesis.

## Materials and methods

### Ethics statement

This study was approved by the Ethics in Research Committee at the Federal University of Goiás (report number 965.662, dated February 26, 2015) and was conducted according to the Ethical Principles for Medical Research Involving Human Subjects of the World Medical Association Declaration of Helsinki, and after a brief explanation about the survey objectives, written informed consent was obtained from all participants.

### Subjects

A total of 117 SAH patients (age range: 22–86 years) and 123 controls (age range: 32–84 years) were enrolled in this study. All patients were undergoing treatment for hypertension (diagnosed as systolic blood pressure ≥ 140 mmHg and/or diastolic blood pressure ≥ 90 mmHg) for at least two years at the League of Hypertension of Faculty of Medicine of Federal University of Goiás. The control group consisted of diabetes-free and normotensive individuals selected from the general population of our region matched to the case group in approximated proportions of sex and age range. Clinical and laboratory data of patients was extracted from their record files. The mean of the second and third pressure registers was used to obtain the systolic and diastolic pressure values. For the control group, it was used recent laboratory data and blood pressure was measured in the time of interview. Data on lifetime, smoking habits, alcohol consumption, general health conditions, medications in use, previous diseases and other anamnesis data were obtained through interviews. All individuals selected for this study were classified as non-white and non-black from the Central Brazil region.

### Genotyping of *ACE* and *ACE2* polymorphisms

The genotyping of ACE I/D polymorphism was carried out by real time PCR (qPCR, with Fermentas Master Mix) followed by melting curve analysis. This method allows identify the I and D alleles by the different melting peaks generated according to the length and nucleotide composition of the insertion and deletion amplicons. It was adopted the protocol developed by Lin et al., 2001 [14] with the following primers: pACE1-F (5′-CATCCTTTCTCCCATTTCTC-3′), pACE2-F (5′-TGGGATTACAGGCGTGATACAG-3′) and pACE3-R (5′-ATTTCAGAGCTGGAATAAAATT-3′), with a modification: the three primers used (two Forward–pACE1-F e pACE2-F—and one Reverse–pACE3-R) were separated in two different tubes of PCR (pACE1-F with pACE3-R and pACE2-F with pACE3-R) to obtain a better differentiation of the melting peaks for I and D alleles. All reactions were performed in duplicate, and all genotypes were confirmed.

The genotyping of ACE2 G8790A polymorphism were performed by restriction fragment length polymorphism PCR (PCR-RFLP, with Invitrogen reagents). It was adopted the protocol established by Benjafield et al., 2004 [24] with the following primers: Forward (5′-CAT GTG GTC AAA AGG ATA TCT-3′) and Reverse (5′-AAA GTA AGG TTG GCA GAC AT-3′), with a modification: the time of incubation with restriction enzyme, Alu I (Invitrogen), was carried out during an overnight period to ensure a complete digestion of the PCR product. After digestion, fragments of 281 and 185 bp identify A allele and a 466 bp band identify G allele. After electrophoresis, bands were visualized on silver nitrate–stained polyacrylamide gels and all genotypes were confirmed.

### Statistical analysis

Categorical variables were analyzed by chi-square (χ^2^) or Fisher’s exact test when necessary. Continuous variables were expressed as media plus standard deviation and analyzed by Student t test in the comparison between case and control groups or by one-way analysis of variance (ANOVA) followed by Bonferroni’s post hoc test in the comparison among three genotypes. To analyze the risk conferred by the genotypes, it was used odds ratio (OR) calculation (with the non-risk allele or genotype considered as reference) with respective 95% confidence intervals (95% CI) and multinomial logistic regression modelling was applied to obtain adjusted odds ratio values (OR^A^). The confounding factors used in logistic regression modeling was age (categorized as elderly [≥ 60 years] and not elderly), BMI (categorized as obese [≥ 30 kg/m^2^] or non-obese), dyslipidemia (considering dyslipemic individuals with triglycerides>150mg/dl and/or LDL-cholesterol>130mg/dl and/or HDL-cholesterol<50mg/dl for female or HDL-cholesterol<40mg/dl for male), smoking habits (considering smokers as individuals that have smoked for at least one year before the diagnosis of HAS) and alcohol consumption (individuals that have the habit of use alcohol at least occasionally). Hardy-Weinberg equilibrium was assessed by the χ2 test. The level of significance adopted was p<0.05. For the ACE2 polymorphism, data for each sex were analyzed separately once ACE2 gene is on the X chromosome.

## Results

General characteristics of the study population can be seen in Table 1. Distribution by sex, age, smoking, alcohol consumption and body mass index (BMI) was similar between case (hypertensive) and control (normotensive) groups, indicating homogeneity for the subsequent analysis. As expected, blood pressure and laboratory data of lipid profile and blood glucose were significantly different between the groups due to the very more altered values for these variables observed in hypertensive patients, reflecting the incidence of type 2 diabetes mellitus (T2DM) [37% of patients] and dyslipidemia (47% of patients), that are comorbidities frequent in these patients. The mean duration of illness was 15.8 ± 10.2 years and the follow-up time was 10.5 ± 7.3 years.

**Table 1:** Characteristics of the study population.

Variables	Case (SAH patients) N = 117	Control N = 123	P
Sex (M/F)	30/87	42/81	0.195
Age (years)	59.65 ± 11.67	57.46 ± 9.70	0.115
BMI (kg/m^2^)	29.27 ± 5.42	28.05 ± 4.84	0.069
SBP (mmHg)	145.86 ± 20.20	122.89 ± 10.37	<0.0001*
DBP (mmHg)	86.05 ± 11.82	75.25 ± 8.09	<0.0001*
Triglycerides (mg/dl)	164.14 ± 85.67	125.76 ± 40.86	<0.0001*
Total Cholesterol (mg/dl)	203.32 ± 47.11	153.48 ± 26.13	<0.0001*
HDL Cholesterol (mg/dl)	45.07 ± 11.05	49.67 ± 9.55	0.001*
Não-HDL Cholesterol (mg/dl)	158.25 ± 47.44	103.81 ± 26.89	<0.0001*
VLDL Cholesterol (mg/dl)	33.65 ± 17.72	25.15 ± 8.17	<0.0001*
Fasting Blood Glucose (mg/dl)	111.37 ± 45.80	87.84 ± 8.62	<0.0001*
Creatinin (mg/dl)	0.97 ± 0.48	1.01 ± 0.21	0.353
Smoking Habits (+/-)	45/72	47/76	1.000
Alcoholism (+/-)	22/95	32/91	0.237
Time of Disease (years)	15.79 ± 10.20	————-	————-
Follow-up time (years)	10.48 ± 7.28	————-	————-

Analysis by T test or chi-square (χ^2^).

*Significant difference between groups (p <0.05). SBP—Systolic Blood Pressure; DBP—Diastolic Blood Pressure. Significance between groups: p<0.05.

Regarding the genotypic frequencies (Table 2), in the case group, the results obtained for *ACE* I/D polymorphism were 20.5% (II), 54.7%(ID) and 24.8% (DD). For *ACE2* G8790A polymorphism, the frequencies were 47.1% (GG), 47.1%(GA) and 5.7% (AA) among female subjects, and allelic frequencies on male subjects were 66.7% (G) and 33.3% (A). For the control group, the frequencies were 27.6% (II), 54.5%(ID) and 17.9% (DD); 55.6% (GG), 35.8%(GA) and 8.6% (AA) and 76.2% (G) and 23.8% (A), respectively. No difference was observed in the distribution of genotypic and allelic frequencies of *ACE* I/D and *ACE2* G8790A polymorphisms between case and control groups (analysis by qui-square test).

**Table 2:** Distribution of genotypic frequencies for *ACE* I/D and *ACE2* G8790A polymorphisms in the study population and a risk analysis for SAH.

	Case	Control					
Genotype	N (%)	N (%)	χ^2^	P^1^	OR (95%CI)	ORA (95%CI)	P^2^
***ACE***	117 (100.0)	123 (100)					
II	24 (20.5)	34 (27.6)	-------	---------	1 (Reference)	1 (Reference)	---------
ID	64 (54.7)	67 (54.5)	0.627	0.428	1.35 (0.72–2.53)	1.45 (0.73–2.90)	0.292
DD	29 (24.8)	22 (17.9)	2.021	0.155	1.87 (0.87–4.00)	1.88 (0.79–4.48)	0.150
ID+DD	93 (79.5)	89 (72.4)	1.297	0.255	1.55 (0.81–2.69)	1.61(0.80–3.02)	0.198
***ACE2*** ***Female***	87 (100.0)	81 (100.0)					
GG	41 (47.1)	45 (55.6)	-------	---------	1 (Reference)	1 (Reference)	---------
AG	41 (47.1)	29 (35.8)	1.427	0.232	1.55 (0.82–2.93)	1.45 (0.76–2.94)	0.293
AA	5 (5.7)	7 (8.6)	0.007	0.935	0.78 (0.23–2.66)	0.83 (0.22–2.86)	0.771
AG+AA	46 (52.9)	36 (44.4)	0.879	0.348	1.40 (0.76–2.58)	1.32 (0.69–2.55)	0.400
**Male**	30 (100.0)	42 (100.0)					
G allele	20 (66.7)	32 (76.2)	-------	---------	1 (Reference)	1 (Reference)	---------
A allele	10 (33.3)	10 (23.8)	0.388	0.534	1.60 (0.57–4.52)	2.16 (0.49–9.54)	0.309

Analysis by chi-square (χ^2^), Odds Ratio calculation (OR) with confidence intervals (95%CI) and multinomial logistic regression to obtain adjusted odds ratio values (OR^A^). P^1^ - p values of chi-square or Fisher’s Exact Test. P^2^ - p values of OR^A^. Significance between groups: p<0.05.

Considering the risk analysis for each gene (Table 2), there was no significant association between the *ACE* I/D and *ACE2* G8790A polymorphisms and the susceptibility to SAH (p > 0.05).

The analysis of the combined genotypes of the two genes (Table 3) revealed an important association for the combination of *ACE* DD genotype with *ACE2* GG genotype (DD/GG) on female subjects, that conferred a significative (p = 0.01) increased risk of 7 times in developing hypertension compared to individuals carrying the II/GG genotype (dominant model for *ACE2* –grouping GA and AA individuals) after adjustment by multinomial logistic regression for confounding factors regarding age (elderly and not-elderly), dyslipidemia (presence and absence), BMI (obese and non-obese), smoking and alcohol consumption. The significative result was maintained for the DD/GG or GA (DD/G_) carriers (OR = 3.6, p = 0.03) in the recessive model for *ACE2* (grouping GG and GA individuals), however the association became smaller than that observed for DD/GG genotype. No significative association was verified among male subjects.

**Table 3:** Distribution of genotypic frequencies for the combination of *ACE* I/D polymorphism and *ACE2* G8790A polymorphism in the study population and a risk analysis for SAH.

	Case	Control					
Genotype	N (%)	N (%)	*χ^2^*	*P^1^*	OR (95%CI)	OR^A^ (95%CI)	*P^2^*
***ACE/ACE2***							
***Female***	87 (100.0)	81 (100.0)					
***ACE/ACE2 Dominant Model***							
II/GG	8 (9.2)	17 (21.0)	-------	---------	1 (Reference)	1 (Reference)	---------
II/GA or AA	10 (11.5)	9 (11.1)	1.143	0.285	2.36 (0.69–8.09)	2.40 (0.62–9.33)	0.205
ID/GG	21 (24.1)	24 (29.6)	0.884	0.347	1.86 (0.67–5.18)	1.74 (0.58–5.22)	0.320
ID/GA or AA	27 (31.0)	19 (23.5)	3.612	0.057	3.02 (1.08–8.42)	2.94 (0.98–8.84)	0.055
DD/GG	12 (13.8)	4 (4.9)	5.601	0.018*	6.38 (1.56–26.10)	7.12 (1.50–33.83)	0.014*
DD/GA or AA	9 (10.3)	8 (9.9)	1.075	0.300	2.39 (0.67–8.51)	1.82 (0.46–7.25)	0.393
***ACE/ACE2 Recessive Model***							
II/GG or GA	16 (18.4)	26 (32.1)	-------	---------	1 (Reference)	1 (Reference)	---------
II/AA	2 (2.3)	0 (0.0)	^#^	0.1617	------------	------	------
ID/GG or GA	46 (52.9)	40 (49.4)	2.68	0.148	1.87 (0.88–3.97)	1.78 (0.79–4.00)	0.163
ID/AA	2 (2.3)	3 (3.7)	^#^	0.9944	1.08 (0.16–7.20)	1.33 (0.18–9.54)	0.779
DD/GG or GA	20 (23.0)	8 (9.9)	7.47	0.013	4.06 (1.45–11.38)	3.57 (1.16–10.95)	0.026*
DD/AA	1 (1.1)	4 (4.9)	^#^	0.640	0.40 (0.04–3.96)	0.41 (0.04–4.36)	0.459
**Male**	30 (100.0)	42 (100.0)					
***ACE*/*ACE2***							
II/G	3 (10.0)	5 (11.9)	-------	---------	1 (Reference)	1 (Reference)	---------
II/A	3 (10.0)	3 (7.1)	^#^	1.000	1.67 (0.19–14.27)	0.58 (0.03–12.20)	0.726
ID+DD/G	17 (56.7)	27 (64.3)	^#^	1.000	1.05 (0.22–4.97)	0.89 (0.10–8.07)	0.920
ID+DD/A	7 (23.3)	7 (16.7)	^#^	1.000	1.67 (0.28–9.82)	3.89 (0.27–56.72)	0.321

Analysis by chi-square (χ^2^) or Fisher’s Exact Test^#^, Odds Ratio calculation (OR) with confidence intervals (95% CI) and multinomial logistic regression to obtain adjusted odds ratio values (OR^A^). P^1^ - p values of chi-square or Fisher’s Exact Test. P^2^ - p values of OR^A^.

*Significant difference between groups (p <0.05).

Old age and dyslipidemia were the variables that presented significative association with SAH in all multinomial logistic regression analyses performed (data not shown) constituting independent risk factors for the disease.

The distribution of the *ACE* and *ACE2* genotypes in the case and control groups was consistent with the expected by the Hardy-Weinberg equilibrium (p > 0.05, Table 4). This result suggests a representative sampling of the study subjects in the research field.

**Table 4:** Test for Hardy-Weinberg equilibrium for *ACE* I/D and *ACE2* G8790A polymorphisms on case (SAH patients) and control groups.

Genotype	Obs.	Exp.	χ^2^ (1 D.F.)	P	Alleles	Frequency
***ACE***						
**Case**						
II	24	26.8	1.079	0.2990	I	0.48
ID	64	58.4			D	0.52
DD	29	31.8				
Total	117	117				
**Control**						
II	34	37.0	1.228	0.2679	I	0.53
ID	67	60.9			D	0.47
DD	22	25.0				
Total	123	123				
***ACE2***						
**Case—Female**						
GG	41	43.5	1.639	0.2005	G	0.71
GA	41	36.1			A	0.29
AA	5	7.5				
Total	87	87				
**Control—Female**						
GG	45	43.7	0.543	0.4611	G	0.73
GA	29	31.6			A	0.27
AA	7	5.7				
Total	81	81				

Analysis by chi-square (χ^2^). Obs.–Observed; Exp.–Expected; DF–Degree of Freedom. Significance between groups: p<0.05.

By the analysis of the influence of *ACE* I/D genotypes on blood pressure and biochemical variables among hypertensive patients (Table 5), it was verified significative increased levels of VLDL-cholesterol on D allele carriers (p = 0.033). Triglycerides, total cholesterol and HDL Cholesterol were close to the level of significance for D allele (p<0.10). By the inclusion of control group to this analysis (case + control groups), increasing the sample size, we could observe that the association with altered lipid profile variables was maintained and reinforced bringing significative increased levels of triglycerides (p = 0.012) and VLDL-cholesterol (p = 0.013) for DD genotype carriers in relation to II genotype (Table 6). Regarding *ACE2* G8790A polymorphism, no significative association was verified for both female (Table 7) and male patients (Table 8).

**Table 5:** Analysis of the influence of *ACE* I/D polymorphism on clinical-laboratory variables among SAH patients.

Variables	II (N = 24)	ID (N = 64)	DD (N = 29)	P IIxIDxDD	P II x [ID+DD]
Sex (M/F)	6/18	16/48	8/21	NS	0.8560
Age (Years)	60.8 ± 11.7	59.2 ± 12.0	59.6 ± 11.1	0.858	0.593
SBP (mmHg)	86.1 ± 12.2	85.9 ± 10.5	86.3 ± 14.4	0.629	0.449
DBP (mmHg)	143.1 ± 18.5	145.7 ± 20.9	148.4 ± 20.3	0.986	0.965
BMI (Kg/m^2^)	29.6 ± 5.5	29.0 ± 5.1	29.7 ± 6.1	0.807	0.758
Triglycerides (mg/dl)	142.8 ± 53.7	159.2 ± 67.2	192.6 ± 128.8	0.086	0.069
Cholesterol (mg/dl)	219.2 ± 52.0	200.0 ± 48.1	197.5 ± 38.5	0.175	0.064
HDL-Cholesterol (mg/dl)	48.8 ± 8.7	45.0 ± 11.3	42.1 ± 11.6	0.092	0.067
Non-HDL Cholesterol (mg/dl)	170.5 ± 53.3	155.0 ± 48.2	155.4 ± 40.1	0.371	0.158
VLDL-Cholesterol (mg/dl)	28.6 ± 10.7	33.1 ± 14.1	39.1 ± 26.5	0.092	0.034*
Creatinin (mg/dl)	0.9 ± 0.2	1.0 ± 0.3	1.0 ± 0.8	0.542	0.535
Fasting Blood Glucose (mg/dl)	104.7 ± 26.2	114.7 ± 52.8	109.2 ± 41.6	0.643	0.438

Analysis by ANOVA (IIxIDxDD), Student t test (II x ID+DD), or chi-square test (χ^2^). NS–non-significative in all comparations.

*Significant difference between groups (p <0.05). SBP—Systolic Blood Pressure; DBP—Diastolic Blood Pressure.

**Table 6:** Analysis of the influence of ACE I/D polymorphism on clinical-laboratory variables among case + control individuals.

Variables	II (N = 58)	ID (N = 131)	DD (N = 51)	P-IIxIDxDD
Sex (M/F)	14/44	40/91	18/33	NS^1^
Age (Years)	58.5 ± 10.2	58.7 ± 11.0	58.2 ± 10.7	0.963
SBP (mmHg)	131.7 ± 16.5	134.1 ± 20.1	136.8 ± 21.6	0.400
DBP (mmHg)	79.8 ± 11.4	80.4 ± 10.7	81.7 ± 13.3	0.672
BMI (Kg/m^2^)	29.3 ± 4.9	28.4 ± 5.1	28.4 ± 5.6	0.547
Triglycerides (mg/dl)	129.5 ± 46.9	141.9 ± 56.6	168.0 ± 105.7	0.012*
Cholesterol (mg/dl)	181.3 ± 50.7	175.4 ± 45.3	179.9 ± 38.6	0.659
HDL-Cholesterol (mg/dl)	49.7 ± 8.7	47.5 ± 11.1	44.8 ± 10.5	0.054
Non-HDL Cholesterol (mg/dl)	131.7 ± 53.0	127.9 ± 46.3	135.1 ± 41.5	0.631
VLDL-Cholesterol (mg/dl)	25.9 ± 9.4	29.0 ± 11.9	33.9 ± 21.8	0.013*
Creatinin (mg/dl)	0.9 ± 0.2	1.0 ± 0.3	1.1 ± 0.6	0.199
Fasting Blood Glucose (mg/dl)	95.6 ± 19.5	100.4 ± 39.7	100.3 ± 33.4	0.655

Analysis by ANOVA (IIxIDxDD), Student t test (II x ID+DD), or chi-square test^1^ (χ^2^). NS–non-significative in all comparations.

*Significant difference between groups (p <0.05). SBP—Systolic Blood Pressure. DBP—Diastolic Blood Pressure.

**Table 7:** Analysis of the influence of *ACE2* G8790A polymorphism on clinical-laboratory variables among HAS female patients.

Variables	GG (N = 41)	GA (N = 41)	AA (N = 5)	P GGxGAxAA	P GG x [AG+AA]
Age (Years)	59.7 ± 13.0	58.9 ± 9.7	55.6 ± 10.2	0.747	0.642
SBP (mmHg)	148.3 ± 23.0	145.1 ± 16.1	136.3 ± 9.5	0.389	0.332
DBP (mmHg)	87.4 ± 12.4	86.1 ± 11.2	81.2 ± 13.6	0.527	0.470
BMI (Kg/m^2^)	30.0 ± 5.6	29.3 ± 5.7	25.6 ± 3.1	0.246	0.346
Triglycerides (mg/dl)	154.4 ± 58.8	155.9 ± 67.4	154.6 ± 83.0	0.994	0.919
Cholesterol (mg/dl)	201.1 ± 53.6	205.3 ± 41.6	229.6 ± 21.6	0.444	0.501
HDL-Cholesterol (mg/dl)	45.3 ± 11.9	48.0 ± 9.5	46.8 ± 8.4	0.525	0.266
Non-HDL Cholesterol (mg/dl)	155.8 ± 52.9	157.3 ± 42.7	182.8 ± 19.9	0.480	0.674
VLDL-Cholesterol (mg/dl)	30.9 ± 11.8	33.1 ± 14.8	30.9 ± 16.6	0.749	0.495
Creatinin (mg/dl)	1.0 ± 0.7	0.9 ± 0.2	0.8 ± 0.1	0.503	0.268
Fasting Blood Glucose (mg/dl)	120.4 ± 58	110.6 ± 45.7	107.4 ± 17.8	0.647	0.354

Analysis by ANOVA (GGxGAxAA), Student t test (GG x [GA+AA]), or chi-square test (χ^2^). NS–non-significative in all comparations. SBP—Systolic Blood Pressure; DBP—Diastolic Blood Pressure. Significance between groups: p<0.05.

**Table 8:** Analysis of the influence of *ACE2* G8790A polymorphism on clinical-laboratory variables among HAS male patients.

Variables	G (N = 20)	A (N = 10)	P
Age (Years)	60.8 ± 14.6	62.6 ± 8	0.7207
BMI (Kg/m^2^)	29.3 ± 5.6	27.3 ± 3.5	0.3019
SBP (mmHg)	149.7 ± 24.9	135.8 ± 13.5	0.1110
DBP (mmHg)	85.4 ± 13	83.8 ± 10.0	0.7230
Triglycerides (mg/dl)	171.1 ± 89.8	228.6 ± 182.9	0.3677
Cholesterol (mg/dl)	194.2 ± 39	209.0 ± 63.8	0.4362
HDL-Cholesterol (mg/dl)	38.9 ± 10.7	43.4 ± 11.8	0.2974
Non-HDL Cholesterol (mg/dl)	155.4 ± 43.4	165.6 ± 62.2	0.6021
VLDL-Cholesterol (mg/dl)	35.1 ± 19.3	45.7 ± 36.6	0.3026
Creatinin (mg/dl)	1.1 ± 0.4	1.1 ± 0.3	0.8105
Fasting Blood Glucose (mg/dl)	97.1 ± 16.2	106.5 ± 32.3	0.4031

Analysis by Student t test. SBP—Systolic Blood Pressure. DBP—Diastolic Blood Pressure. Significance between groups: p<0.05.

## Discussion

The results obtained in this genetic association study indicate that *ACE* I/D and *ACE2* G8790A polymorphisms individually are not associated with the risk to SAH development, but the combination of *ACE* DD genotype with *ACE2* G allele conferred an important susceptibility to the disease on female gender (OR = 3.6, p = 0.03), with a stronger susceptibility on DD/GG carriers (OR = 7.1, p = 0.01). These results were confirmed after adjusting by age, BMI, dyslipidemia, smoking and alcohol consumption, and, of these, age > 60 years and presence of dyslipidemia were the variables that behaved as independent risk factors for hypertension in the analyzed sample. Besides that, a contribution of D allele of *ACE* I/D polymorphism to dyslipidemia was also verified by alterations on VLDL-cholesterol (D allele on case group, p = 0.03; DD genotype in all individuals analysis, p = 0.01) and Triglycerides levels (DD genotype in all individuals analysis, p = 0.01).

Considering other studies carried out in the Brazilian population, Bonfim-Silva *et al*. (2016)[25] have observed no significative association for the *ACE* I/D polymorphism and hypertension evaluating African-Brazilian and Caucassian-Brazilian subjects of the Northeast region of Brazil. Regarding frequencies distribution, the proportion of I and D alleles were slightly different from the distribution observed in this study with a higher proportion of the D allele (around 65% *versus* 50% in this study) resulting in a greater frequency of individuals with DD genotype (around 40% *versus* 20% in this study). That difference can be attributed to the heterogeneity in ethnical-racial composition among regions of Brazil and to the specific racial group evaluated.

In another Brazilian study, Sakuma *et al*. (2004) [26] have evaluated *ACE* I/D polymorphism association with coronary artery disease in hypertensive and normotensive African-Brazilian subjects from a state of the Central Brazil region (Mato Grosso do Sul) and they have obtained allelic frequencies very close to the observed in this study (around 50% for each allele). For *ACE2* polymorphism, no other study was observed in the Brazilian population.

Studies carried out in other populations have brought conflicting results for each polymorphisms evaluated and some of them have found absent or limited association for *ACE* [27–30] and *ACE2* [24,31,32] polymorphisms, as in this study. Nevertheless the risk allele for hypertension indicated by robust metanalysis studies point out the D allele (or DD genotype)[16] of *ACE* I/D polymorphism; while for *ACE2* G8790A polymorphism the findings are more controversial with the great majority of the studies conducted in China been pointed out the A allele (or AA genotype) as the susceptibility profile [33,34].

Only two other studies [35,36], conducted in different ethnic groups of the Chinese population, addressed *ACE* and *ACE2* polymorphisms together in susceptibility to hypertension, but none of them explored the interaction or combination between the two genes. The former found no association for both polymorphisms; the second observed a weak association only for *ACE2* (A allele and AA genotype).

In relation to the genotypic and allelic frequencies, considering the control group, the distribution found for *ACE* I/D polymorphism (30% II, 50% ID and 20% DD with 50% for each allele) approximate the proportion of 1:2:1 already described on white people [37] and has congruency with a robust American population study [27] been closer to the subgroup of Non-Hispanic whites. By contrast, the proportions observed in African descendants evidence a higher frequency of D allele (around 60%) [37,38], while, in Oriental population, the frequency of I allele is usually higher, reaching 60% in Chinese [35] and 70% in Japanese [28].

For the *ACE2* G8790A polymorphism, most of the studies were conducted in China [39] and the frequencies observed in the Chinese population indicate a higher proportion of A allele (around 50% *versus* 25% in this study) [32,35,36] with a greater proportion of AA genotype (around 30% *versus* 9% in this study).

It is of great importance to bring up for discussion that *ACE* gene presents an enormous quantity of polymorphisms, with a further 77 variant sites being already identified and particularly in the region surrounding the *Alu* sequence (intron 16) there are 17 SNPs that have been linked with high degree of disequilibrium with *ACE* I/D polymorphism [13,40]. Particularly, Zhu et al. (2001) [41] have identified a SNP (A11860G) in exon 17 of the gene, as having the most significative effect in the enzyme concentration (19% of the total variance), despite the I/D polymorphism (in the adjacent intron), that had no effect. Taken together, those findings allowed raise the hypothesis that the specific combinations (haplotypes) formed with the I and D alleles can be the real responsible for the associations verified in this and other studies involving the *ACE* I/D polymorphism, which can function as a genetic marker for the variation flanking it.

Regarding ACE2, once G8790A polymorphism occurs on the beginning of the intron (fourth base of the third intron), it is possible that this SNP can cause changes in mRNA montage by alternative splicing and affects gene expression [34,39], however we could observe a scarcity of studies in this matter. Strong linkage disequilibrium of G8790A polymorphism with other two or three intronic SNPs in ACE2 gene have already been described [24,32].

The results of this study point out that the addition of ACE2 polymorphism in combination with that genetic marker can improve the susceptibility analysis and the strong association verified, in fact, can carry the weight of about 20 polymorphic sites of the *ACE* genes in combination. The findings obtained are unprecedented and corroborate the theory about the complex genetic nature of hypertension, that relies on the interaction of several polymorphisms in diverse genes [42], with small contribution of each individual polymorphism and indicates that the combination of genetic polymorphisms of angitensinases may play a role in hypertension susceptibility.

Literature data indicate that *ACE* DD genotype leads to higher levels of activity of this enzyme [15,22] which has predominantly prohypertensive activity. In relation to ACE2, which presents predominantly antihypertensive activity [23], it was found only one functional assessment study of this enzyme in correlation with G8790A polymorphism indicating a lower activity in GG, intermediate in GA and higher in AA genotype [43]. The results obtained in this study indicate that the combination of genetic polymorphisms of *ACE* and *ACE2* may contribute to the development of hypertension, with the DD/G_ combination being the susceptibility profile. This finding in association with the referred functional study allows us to launch the hypothesis that an increased activity of ACE in conjunction with reduced ACE2 activity would explain the increased susceptibility to hypertension in the DD/G_ profile.

The associations of *ACE* D allele or DD genotype with alterations on lipid profile variable are consistent with other studies on hypertensive patients [44] and CVD patients [19,45] and have also been observed in T2DM patients [46], been suggested the involvement of overactivated RAAS (by the increased activity of ACE on D carriers) in dyslipidemia as a possible underlying mechanism [46], as it has been well documented the role of ACE inhibitors in improving atherosclerosis and insulin resistance [47], nevertheless this issue still remains little explained in the literature and deserves more clarification.

We achieved equal distribution by sex, average age, BMI, smoking and alcohol consumption between case and control groups and the results obtained in the logistic regression analysis allowed the identification only of old age (>60 years) and dyslipidemia as independent risk factors for hypertension in the studied population, besides the combined polymorphisms of ACE and ACE2. This indicates that the monitoring of older age groups and the monitoring and control of dyslipidemia are important strategies for the prevention of hypertension in public health, and individuals with the DD/G_ genotype would constitute a priority attention group if genotyping of these polymorphisms was incorporated as an early diagnostic tool after confirmation with more robust studies.

The genetic susceptibility to SAH conferred by the combined genotypes of ACE and ACE2 (DD/G_), that were not observed in the analysis of ACE and ACE2 polymorphisms separately indicates a synergistic effect of the “risk variants” of the two genes in the disease pathogenesis and corroborate the literature data about the function of these enzymes as two arms that work together to control and maintain pressure levels [7,48].

Synergism among genetic variants in other components of the RAAS has already been reported [49], nevertheless, to our knowledge, this is the first study exploring the effect of the combination of ACE and ACE2 polymorphisms on hypertension susceptibility. We encourage other researchers to investigate the interaction effects of ACE and ACE2 polymorphisms on hypertension and CVD given the results obtained in this study, besides that, the known counterbalancing role of those two enzymes in RAAS system become necessary that evaluation to a proper understanding of pressure dysregulation mechanisms.

As limitations or complementation of this study, we must highlight that researches involving a larger sample size and the determination of ACE and ACE2 activities in plasma could bring more information about the interaction verified. In this regard, the development of an assay to simultaneous detection/dosage of ACE and ACE2 would be an important tool to advance in this area. It is important to underline that, in this study, T2DM hypertensive patients constituted only 37% of the patients and a study with a greater sampling would be designed to allow the stratification of HAS patients according to T2DM status in different groups. Finally, the analysis of the influence of combined ACE and ACE2 polymorphisms in treatment response profile on hypertension and CVD can be of great help for personalized medicine if the findings of this study were confirmed by other researchers.

## Conclusions

The results obtained in this study have shown that elderly age, dyslipidemia and the combination of genetic polymorphisms of *ACE* and *ACE2* were independent risk factors for hypertension in the sample analyzed. The findings achieved suggest that individuals with the DD/G_ genotype and more pronouncedly DD/GG individuals present a greater susceptibility to SAH development, which after being confirmed in new researches involving a greater sampling, could be used as an early diagnosis criterion for the disease. The association of the ACE D allele with changes in the lipid profile of the patients indicates that this can be a marker of poor prognosis in the evolution of the disease.

The findings achieved in association with the literature data allows us to hypothesize that DD/G_ carriers would have an increased activity of ACE (DD genotype) in conjunction with reduced ACE2 activity (G allele), since ACE D allele have been associated with increased enzyme activity [15,22] and ACE2 G allele seems to cause a reduction in the activity of this enzyme [43] that needs to be more investigated.

Altogether the results obtained suggest that the application of the combination analysis to genetic association studies involving candidate genes with known physiological interplay can be more elucidative in stabilish a genetic susceptibility profile on multifactorial conditions as hypertension than the study of the isolated genes only.
